# Assessment of physical literacy in 8- to 12-year-old Pakistani school children: reliability and cross-validation of the Canadian assessment of physical literacy-2 (CAPL-2) in South Punjab, Pakistan

**DOI:** 10.1186/s12889-024-19185-3

**Published:** 2024-06-28

**Authors:** Syed Ghufran Hadier, Yinghai Liu, Liu Long, Syed Muhammad Zeeshan Haider Hamdani, Haris Khurram, Syed Danish Hamdani, Shaista Shireen Danish, Syeda Urooj Fatima

**Affiliations:** 1 School of Physical Education, Shanxi University, Taiyuan, Shanxi 030006 China; 2 Department of Physical Education, Suzhou University, Suzhou, Anhui 234000 China; 3 Faculty of Social Science, Department of Sports Sciences, Bahauddin Zakariya University, Multan, Punjab 60800 Pakistan; 4 Department of Mathematics and Computer Science, Faculty of Science and Technology, Prince of Songkla University, Pattani Campus, Pattani, Thailand; 5 Department of Sciences and Humanities, National University of Computer and Emerging Sciences, Chiniot-Faisalabad Campus, Faisalabad, Pakistan; 6 School Education Department, Multan, South Punjab 60800 Pakistan; 7 Department of Sports Sciences, Bahauddin Zakariya University, Multan, Punjab 60800 Pakistan; 8 Department of Physical Education, Government College University Faisalabad, Punjab, 38000 Pakistan; 9 Division of Olympic Sports, China Swimming College, Beijing Sport University, Beijing, China; 10 Division of Sports and Health, School of Sports Science, Department of Exercise Physiology, Beijing Sport University, Beijing, China; 11 School of Exercise and Health, Shanghai University of Sport, Shanghai, China

**Keywords:** Urdu translation, Reliability, Cross-cultural adaptation, CAPL-2, Pakistani children, Confirmatory factor analysis

## Abstract

**Background:**

The increasing prevalence of physical inactivity, declining fitness, and rising childhood obesity highlight the importance of physical literacy (PL), as a foundational component for fostering lifelong health and active lifestyle. This recognition necessitates the development of effective tools for PL assessment that are applicable across diverse cultural landscapes.

**Aim:**

This study aimed to translate the Canadian Assessment of Physical Literacy-2 (CAPL-2) into Urdu and adapt it for the Pakistani cultural context, to assess PL among children aged 8–12 years in Pakistan.

**Method:**

The Urdu version of CAPL-2 was administered among 1,360 children aged 8–12 from 87 higher secondary schools across three divisions in South Punjab province, Pakistan. Statistical analysis includes test-retest reliability and construct validity, employing confirmatory factor analysis to evaluate the tool’s performance both overall and within specific subdomains.

**Results:**

The Urdu version of CAPL-2 demonstrated strong content validity, with a Content Validity Ratio of 0.89. Confirmatory factor analysis supported the four-factor structure proposed by the original developers, evidenced by excellent model fit indices (GFI = 0.984, CFI = 0.979, TLI = 0.969, RMSEA = 0.041). High internal consistency was observed across all domains (α = 0.988 to 0.995), with significant correlations among most, excluding the Knowledge and Understanding domains. Notably, gender and age significantly influenced performance, with boys generally scoring higher than girls, with few exceptions.

**Conclusion:**

This study marks a significant step in the cross-cultural adaptation of PL assessment tools, successfully validating the CAPL-2 Urdu version for the Pakistani context for the first time. The findings affirm the tool’s suitability for assessing PL among Pakistani children, evidencing its validity and reliability across the Pakistani population.

**Supplementary Information:**

The online version contains supplementary material available at 10.1186/s12889-024-19185-3.

## Background

Global public health faces significant challenges due to physical inactivity, poor fitness, and childhood obesity [1, 2], which are intricately linked to non-communicable diseases including heart disease, diabetes, and certain cancers, thereby diminishing quality of life and imposing economic burdens on healthcare systems [3, 4]. In Pakistan, a concerning trend places it 10th globally for obesity prevalence, with approximately 50% of the population being overweight or obese [5], and projections indicate that 5.4 million school-aged children will be obese by 2030 [6]. This alarming situation is largely attributed to physical inactivity and sedentary lifestyles [7], characterized by increased screen time and caloric intake against a backdrop of reduced physical activity, which not only predisposes children to obesity but also impairs their motor skills [8–10]. Against this backdrop, physical activity (PA) remains crucial for children’s physical development, enhancing both gross and fine motor skills [11].

Physical literacy (PL) has emerged as a critical concept within the health and wellness field, as a potential approach to counteracting sedentary behavior, childhood obesity, and, poor fitness including fundamental motor skills (FMS) deficiencies across developed and developing countries [12]. Physical literacy is defined by the International Physical Literacy Association as “the motivation, confidence, physical competence, knowledge, and understanding to value and take responsibility for engagement in physical activities for life” [13], PL represents a holistic pathway to health and fitness [14]. The global research community is actively investigating PL-focused interventions aimed at increasing physical activity levels, promoting healthy weights, and enhancing fitness [15].

Emphasizing the importance of developing PL from an early age [16], especially among school children aged 8–12, recent studies underscore the critical role of PL in fostering an appreciation for physical activity and encouraging a healthy lifestyle [17–19]. Developing PL skills, which encompass FMS, such as running, jumping, throwing, and catching, is crucial for engaging in a variety of physical activities and sports [20]. Such expertise, coupled with an appreciation and awareness of physical activity’s benefits, lays a foundation for sustaining active and healthy lifestyles into adulthood [21, 22].

The emphasis on acquiring Physical Literacy knowledge during pivotal developmental stages is crucial for fostering physical, social, and emotional well-being in children. This approach addresses public health issues stemming from inactivity and obesity [23]. There is a consensus among scholars on the need for empirical research to deepen our understanding of PL [24], including its evaluation across various populations [25], to construct a comprehensive framework [26] and develop robust assessment tools for a holistic evaluation of PL [27, 28]. PL’s foundational role in encouraging lifelong engagement in physical activities [29] highlights its potential to significantly enhance long-term health, satisfaction, and success [30, 31].

However, despite the PL-centric programs and interventions [32], the development of a standardized method for measuring PL remains a challenge [33]. This highlights the urgent need for effective assessment tools to monitor progress and evaluate intervention outcomes [34]. The literature emphasizes the necessity of developing valid and reliable tools for PL assessment to enable practitioners and researchers to understand individual PL levels across its various domains, thereby tailoring more impactful interventions [35–37]. To date, several tools have been developed to ensure a comprehensive measurement of PL, including (a) the Canadian Assessment of Physical Literacy (CAPL-1 and CAPL-2) [38], (b) CS4L’s Physical Literacy Assessment for Youth (PLAY) [39], (c) the Australian Physical Literacy model (PLM) [40], and (d) Physical and Health Education (PHE) Canada’s Passport for Life [41]. While each tool offers unique insights, they also possess limitations, such as limited validation in diverse cultural contexts and some may not evaluate PL in all aspects, pointing to the necessity for ongoing refinement and validation to ensure their efficacy across different settings, except the CAPL-2.

The CAPL-2 distinguishes itself through the incorporation of philosophical and conceptual frameworks, facilitating improved assessment of PL [31, 42]. Its construct validity is vital for ensuring the CAPL-2’s effectiveness in capturing PL’s multifaceted nature, which encompasses physical, cognitive, emotional, and social dimensions. The tool’s validation across diverse child populations from China, Greece, Denmark, Iran, and Spain highlights its cultural adaptability and potential for global use [43].

Although other protocols such as PLAY, the Australian PLM, and PHE Canada’s tools offer certain advantages, the CAPL-2 stands out for its holistic assessment approach, flexibility across ages, and dynamic scoring system. This positions it as the tool of choice for both researchers and educators aiming for an in-depth understanding of PL [39]. Longmuir emphasized the necessity for valid and reliable PL assessment methods and called for the validation of tools across different populations [39, 44]. CAPL-2 is particularly recognized for its rigorous international psychometric testing, reliability, and adaptability across various regions, including East Asia and South Africa, affirming its utility in diverse educational and cultural settings [45, 46]. Originally introduced in 2013, CAPL Version 1’s initial findings were published in 2015 [47]. CAPL Version 2, which incorporated evidence-based modifications, underwent further validation in Canada and was released in 2018 [48]. Table 1 provides an overview of studies on the CAPL’s reliability and validity, supporting its credibility and effectiveness in assessing PL.

**Table 1:** Summary of previous studies

Name	Language	Validity	Reliability
Li et al., (2020) [49]	Chinese	CFA (0.94), TLI (0.90), RMSEA (0.04, (90% CI (0.024–0.062)	ICC = PC = 0.71, DB = 0.72, K&U = 0.52, M&C = 0.82
Dania et al., (2020) [17]	Greek	CFA (0.906), TLI (0.873), RMSEA (0.038, [90% CI (0.030, 0.054)	ICC = PC = 0.62, DB = 0.41, K&U = 0.55, M&C = 0.68
Elsborg et al.,(2021) [16]	Danish	CFA (0.898), TLI (0.853), RMSEA (0.080, (90% CI 0.071–0.090), SRMR = 0.048	NA
Pastor-Cisneros et al., 2022 [50]	Spanish	NA	ICC = PC = 0.88, DB = 0.97, K&U = 0.72, M&C = 0.087
Valadi & Cairney (2022) [51]	Persian	CFA (0.90), TLI (0.90), RMSEA (0.05)	ICC = PC = 0.92, DB = 0.92, K&U = 0.92, M&C = 0.90

*Note*: CFA: Confirmatory factor analysis, TLI: Tucker-Lewis Index, RMSEA: root mean square error of approximation, SRMR: Standardized Root Mean Square Residual, NA; not available

Promoting Physical Literacy among children in Pakistan is an urgent need, yet no assessment tool is currently available to assess PL. This gap necessitates the development or adaptation of an existing comprehensive PL assessment tool for the Pakistani context. The CAPL-2 has emerged from a recent systematic review as the only holistic tool effectively measuring PL [52], with its validity and reliability confirmed in various international contexts [43]. Given its successful validation across diverse populations, it suggests its appropriateness for use in Pakistan, where it has the potential to guide effective PL interventions.

This research aims to address three main objectives. Firstly, this study aimed to translate into Urdu and culturally adapt the CAPL-2 to the Pakistani context. Secondly study aimed to evaluate the PL of school children aged 8–12 in South Punjab, providing critical insights for policymakers and intervention strategies. Finally, the study established the reliability and validity of the adapted CAPL-2 tool in accurately measuring PL among the Pakistani population. Achieving these objectives will enable precise PL assessment in Pakistan, supporting meaningful comparisons within the country and internationally.

## Methods

### Study design and sampling

The current study utilized cross-sectional data to examine the validity and reliability of the CAPL-2 translated into Urdu. Further study employed a stratified random sampling method to obtain a representative sample from South Punjab’s population, considering geographic diversity and representation across all strata. The population was divided into three strata based on the divisions of South Punjab: Multan, Bahawalpur, and Dera Ghazi Khan. Our research concentrates on the South Punjab province, a region overlooked in child health, fitness, and physical literacy studies, to bridge the significant knowledge gap. This area, distinguished by its demographic diversity, largely resulting from migration, and strategic geographical significance, encounters distinct health and education challenges. By exploring Southern Punjab, we aimed to reveal these unique issues, offering insights for crafting more effective child health interventions and PL strategies relevant both locally and in similar contexts globally. Addressing the region’s lack of data, our study aims to stimulate further research and inform policy making, aiming for interventions that accurately meet the local population’s specific needs. This effort not only deepens the academic understanding of child health, physical fitness, and PL in under-researched regions but also advocates for a broader, more inclusive approach to research, thus promoting fair progress in global health and education.

**Fig. 1:**
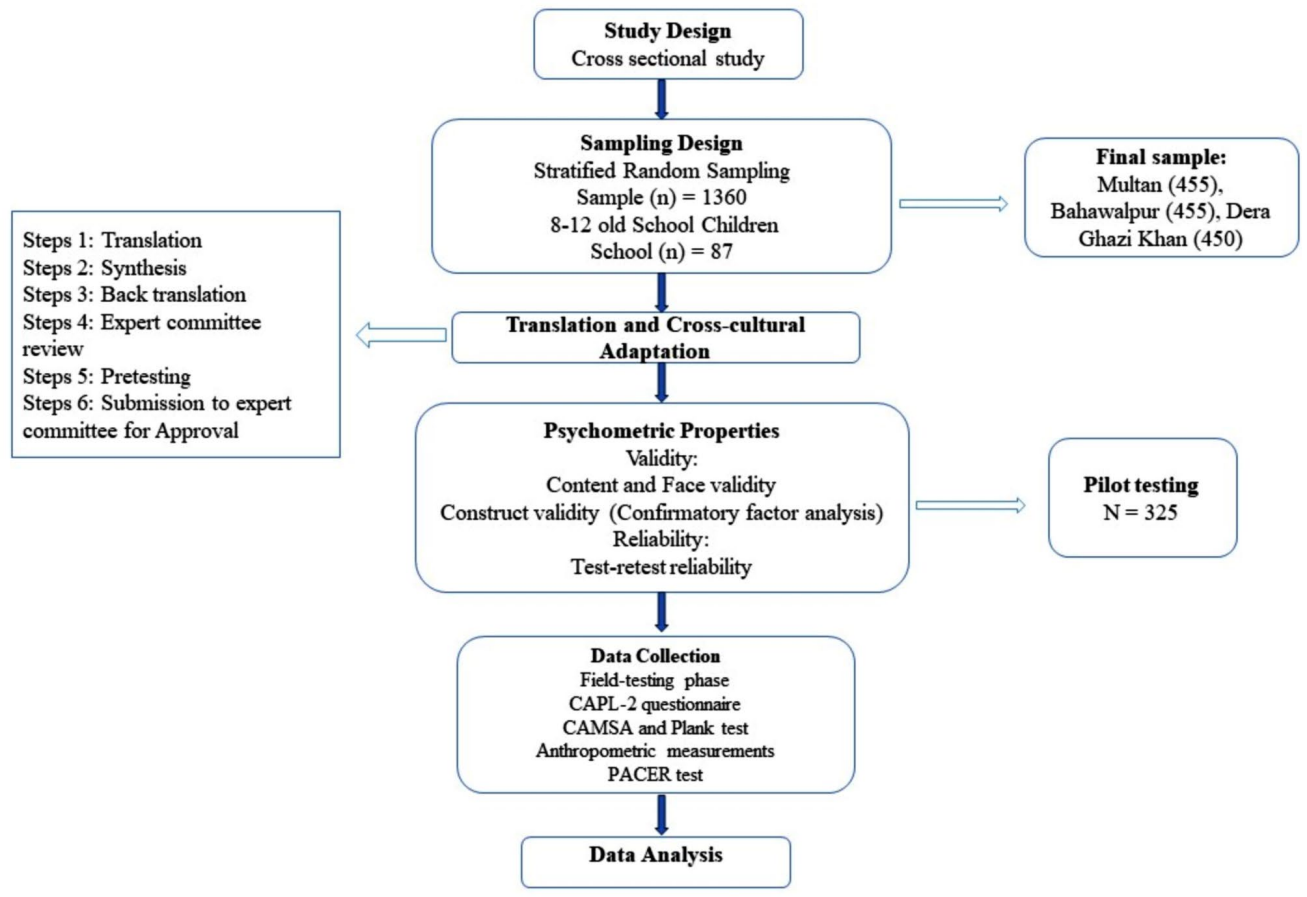
Flow diagram of the study

A total of 1360 students were included in the sample, as determined by Cochran’s (1977) formula: $n=\frac{{\left(Z\right)}^{2}PQ}{{e}^{2}}\times D$, widely used for determining the appropriate sample size [53, 54]. The sample size (n) was calculated as follows: $n=\frac{{\left(1.96\right)}^{2}\left(0.234\right)\left(0.77 \right)}{{\left(0.05\right)}^{2}}\times 5=1359.8 \approx 1360$. In this formula, Z represents the standard normal distribution, which is 1.96 at a 5% significance level. The expected proportion (P) is 0.234, while Q is 0.77 (1 - P). The level of precision e^2^ is 0﻿.02545﻿, and D is the design effect, which is set to 5. A total of 1360 students were selected from 87 higher secondary schools across three strata using an equal allocation method, ensuring representation from all strata [55–57]. From each school’s list of students aged 8–12, 16 were chosen randomly [58]. During the field-testing phase, 11% of the initial participants could not complete all tests, necessitating the replacement of 150 participants with new samples from the same age group and schools. The final sample consisted of 455 participants from Multan, 455 from Bahawalpur, and 450 from Dera Ghazi Khan. This reliable approach, which has been employed in previous studies [43, 59], is likely to produce valid results as a robust sampling method for South Punjab’s population. The study design is detailed in Fig. 1.

### Ethics approval

This research was ethically approved by the Shanxi University in 2020 (Reference: SXULL201912), adhering to the Declaration of Helsinki guidelines. Further ethical clearances were obtained from Pakistani authorities, including Bahauddin Zakariya University, Multan (Reference: 374/UREC/2020), and the South Punjab Department of School Education (Reference: 2189/GB). Participation was voluntary, with minors providing written consent from parents or guardians. The study also received permission to use the CAPL-2 questionnaire from the HALO research group. Following these approvals, we commenced data collection and study procedures.

### Canadian assessment of physical literacy-2 tool

**Fig. 2:**
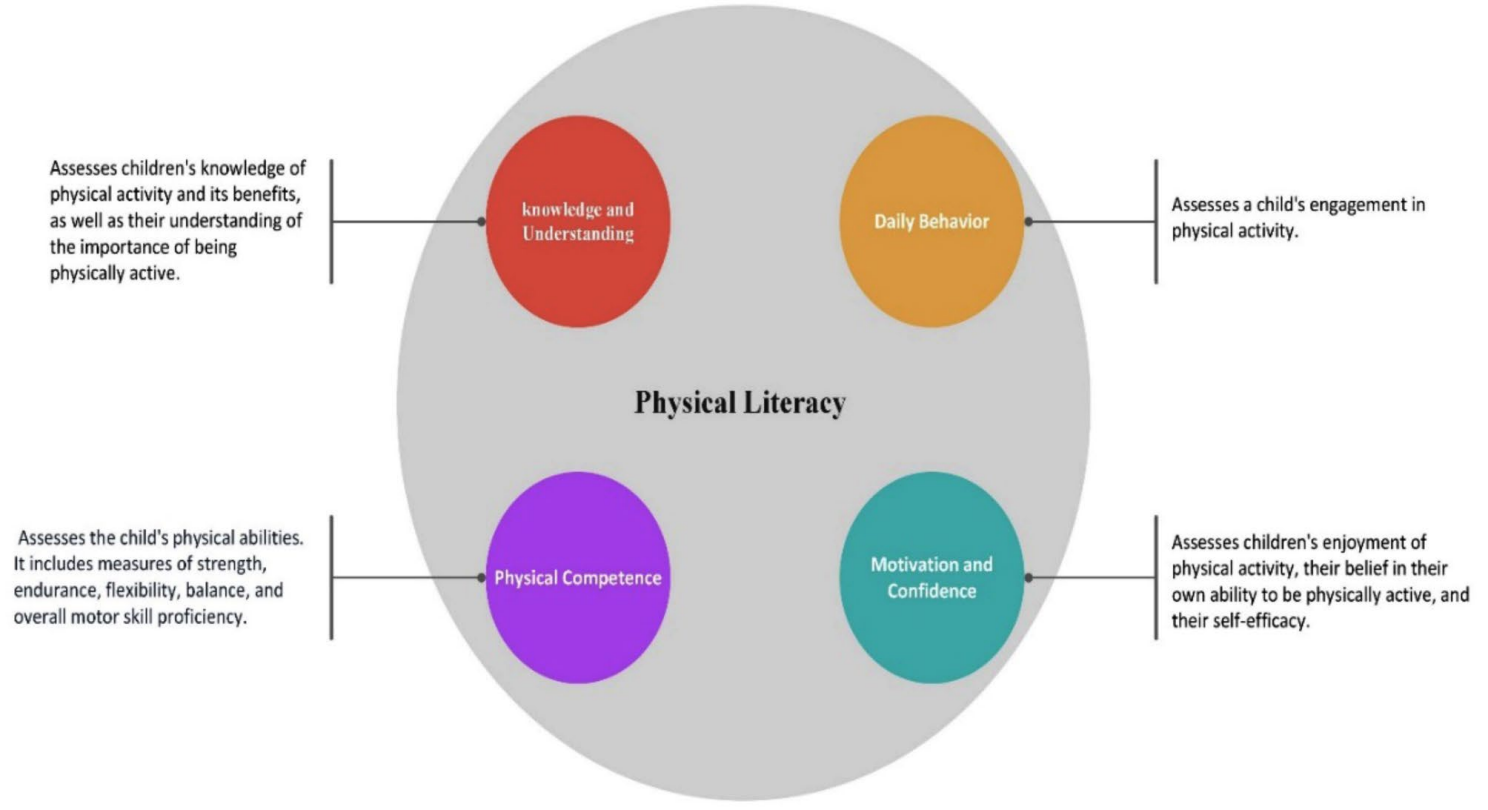
Core domains of PL adapted from CAPL-2 manual HALO (2017) [60]

Introduced in 2015 and subsequently validated across multiple countries [30, 48], the CAPL-2 has served as a robust evaluative tool accurately designed to measure the multifaceted nature of PL in children across four domains: Physical Competence (PC), Motivation and Confidence (M&C), Knowledge and Understanding (K&U), and Daily Behavior (DB) (see Fig. 2). Despite the complex nature of PL, these domains are not discrete; they intersect and influence one another. For example, understanding the benefits of PA may shape an individual’s motivation, and DB often reflects inherent physical competencies.

The interconnection within domains of the CAPL-2 tool extends beyond a mere structural feature, offering significant advantages. It provides a comprehensive view of a child’s physical literacy, closely mirroring the real-world complexity of human physical activity and its priorities. The interaction between domains allows for a nuanced analysis, where insights into one domain can explain aspects of another. This is particularly beneficial for designing integrated intervention strategies. For example, improving a child’s K&U could indirectly enhance their M&C, positively impacting their daily behavior and physical competence. Such a holistic approach is valuable for educators and trainers, emphasizing that enhancements in any domain can lead to overall physical literacy improvement.

The CAPL-2 offers a comprehensive evaluation of physical literacy by integrating objective assessments, self-report questionnaires, and physical activity monitoring [42]. The M&C domain explores an individual’s enthusiasm and self-assurance in engaging with physical activities, encompassing a range of movements and skills. Meanwhile, the PC domain focuses on the acquisition and application of fundamental movement skills in diverse activities. The K&U domain examines one’s comprehension of PA benefits, risks of inactivity, and the intricacies of various sports, including rules and strategies. Lastly, the DB domain quantifies participation in various PA, highlighting their role in promoting health, well-being, and enjoyment.

The CAPL-2 produces a composite score for each domain, ranging from 1 to 100 [48], and offers domain-specific scores for a targeted assessment of areas for improvement. These scores are segmented into four performance levels tailored for diverse age and gender groups: (a) beginning, indicating that children require significant support to improve their PL; (b) progressing, indicating typical performance in PL domains below the recommended level; (c) achieving, indicating that children perform at recommended levels for optimal health benefits; and (d) excelling, indicating that children perform substantially above the recommended levels [60].

The DB domain assessed daily physical activity through objective and subjective methods. The objective method utilizes a pedometer (DigiWalker SW-200, Yamax Corporation, Tokyo, Japan) to measure daily step counts for at least 10 h a day for seven consecutive days, resulting in a composite score ranging from 1 to 25. Statistical data imputation estimates missing or invalid data [60]. The subjective method involves self-reported PA, with children reporting the number of days they engaged in at least 60 min of moderate-to-vigorous physical activity (MVPA) that increased their heart rate. This score ranges from 1 to 5 and is obtained from Sect. 3, item 6 of the CAPL-2U questionnaire [58]. Combining both methods provides a comprehensive assessment of the DB domain, resulting in composite scores ranging from 1 to 30. Further process details are available in a previous study [43].

Children’s physical competence was assessed using the CAPL-2 protocol, consisting of three physical tests scored between 1 and 30 [48]. The first test is the Progressive Aerobic Cardiovascular Endurance Run (PACER), which measures aerobic fitness by having children run back and forth between 20-meter lines marked with cones. The second test is the plank isometric test, assessing muscular strength and endurance by recording the maximum time a child can maintain their body weight on their forearms and toes while maintaining a straight line. The third test, the Canadian Agility and Movement Skill Assessment (CAMSA), assesses fundamental motor skills, agility, combined skills, and complex skills. This test comprises 14 tasks: running, catching, throwing, skipping, sliding, and hopping. Each skill is scored as 1 for correctly performed skills and 0 for not correctly performed skills. The total CAMSA score ranges from 1 to 28, and composite test scores are obtained by dividing the time and skill scores by 2.8, resulting in a score range from 1 to 10. The scores of all three tests are combined to obtain a composite domain score ranging from 1 to 10.

The motivation and confidence domain assesses a child’s affective-motivational and cognitive-confidence aspects, which the self-reported CAPL-2 questionnaire measures. The questionnaire comprises four constructs and 12 items, with scores ranging from 1 to 30, including three items for each construct. The first construct of the M&C domain, predilection, evaluates a child’s interest in and enjoyment of physical activity. The second construct, adequacy, measures a child’s perception of their physical activity level and sense of responsibility for their well-being and health. The third construct, perceived competence, assesses a child’s belief in their ability to perform physical activities. Finally, the fourth construct, intrinsic motivation, measures a child’s internal drive to engage in physical activity for enjoyment rather than external rewards or pressure.

The knowledge and understanding domain assesses children’s understanding of health, fitness, and PA concepts through the CAPL-2 questionnaire’s K&U section. It examines physical competence, daily activity routines, muscular endurance, and cardiovascular fitness concepts. This domain has a total of ten points and consists of five questions. The first four questions are multiple-choice, each valued at one point, and the last requires participants to fill in the blank and is worth six points. Scoring for this domain is based on correct and incorrect responses. Each question receives either 1 point for a correct answer or 0 points for an incorrect response. The domain score is calculated by adding the scores of all the questions.

### Translation and cross-cultural adaptation

The CAPL-2 questionnaire and other testing material underwent a translation and cross-cultural adaptation process, which adhered to the guidelines established by Beaton et al. [61] to ensure validity and reliability. This process included six stages: first, the translation of the materials; second, synthesis of the translated materials; third, a back translation to verify accuracy; fourth, an expert committee review of the quality of the translations; fifth, pre-testing to identify any issues or inconsistencies with the materials; and sixth, submission of documentation of the adaptation process to the expert committee for appraisal. Following these stages ensured a valid and reliable translation and adaptation of the CAPL-2 questionnaire and other testing material detailed in previous study [58].

In Stage 1, two proficient bilingual translators, native in Urdu and with advanced English proficiency (having completed postgraduate degrees in English), were independently chosen to translate the CAPL-2 manual. They prepared separate translations, T1 and T2, without knowledge of each other’s work. In Stage 2, they collaborated on a consolidated Urdu translation, with assistance from a third mediator possessing native Urdu proficiency and exceptional English skills. This culminated in the T3 Urdu version. Stage 3 involved a three-step back translation to verify content validity. Initially, the T3 Urdu version was back translated into English, yielding the BT0 English version. Subsequently, it was translated back into Urdu by two different native translators, resulting in BT1 and BT2 Urdu versions. Lastly, a synthesized BT3 Urdu version was produced by a native Urdu expert in sports science with proficient English skills, ensuring translation consistency and accuracy.

In Stage 4, an expert panel comprised ten reviewers, including five professors, three PhD scholars from various universities’ sports science departments, and five physical education teachers from higher secondary schools. The panel reviewed the T3 Urdu, BT0 English, and BT3 Urdu versions and their respective reports. They suggested modifications, which were incorporated into the final BT3 Urdu version. After one final review, the panel reached a consensus and deemed the CAPL-2UQ version suitable for pilot testing. The content validity of the items was assessed using the Content Validity Ratio (CVR). Items with a CVR score of 0.78 or higher were deemed acceptable, while those below this threshold were subject to modification [62].

In Stage 5, pilot testing was conducted to ensure the feasibility, content, and face validity of the CAPL-2UQ and other testing materials. The pilot testing involved 35 children aged 8 to 12 years and 10 physical education teachers from different higher secondary schools in Multan City. Participants provided feedback on any difficulties or ambiguity in translation, and both PE teachers and students were consulted to ensure suitability. The same group of students underwent a physical competence domain test, which they could understand and perform after the examiner demonstrated them and allowed two trials with verbal instructions. The expert committee reviewed the feedback and made appropriate changes to finalize the CAPL-2UQ version for use in the main study. The researcher gathered feedback from both students and PE teachers to ensure the questionnaire’s feasibility and content validity, and only minor changes were suggested based on overall positive feedback.

During Stage 6, we assessed the CAPL-2U questionnaire and related test manuals for translation and content validity. The principal investigator presented the finalized version, pilot testing reports, face validity reports, and translations to an expert committee for review. The committee thoroughly evaluated the questionnaire, considering all stages of the translation process, and expressed satisfaction with its content validity, only suggesting minor changes. To enhance understanding, we added images to the questionnaire, provided video lectures for physical fitness tests, and increased the number of timed and scored trials from two to three. This change was implemented because the physical fitness tests were new to the children in the south Punjab population, and it allowed them more opportunities to perform the tests accurately. The committee approved the CAPL-2UQ version for further testing upon completing the final review, confirming that it met the standards of content validity and was suitable for use in the main study.

### Psychometric properties

Content and Face Validity: The HALO study group formally allowed the translation of the CAPL-2 manual into Urdu via email confirmation. The current study adhered to Beaton et al.’s [61] translation and cross-cultural adaptation guidelines to ensure accuracy. After completing the initial four steps, pilot testing was conducted in the fifth stage to assess the feasibility, content, and face validity of the CAPL-2UQ and other test materials. In the sixth stage, an expert committee of five members evaluated the Content Validity Ratio (CVR) of the translated CAPL-2UQ to ensure its readability, usability, and comprehensibility for the Pakistani population.

Construct validity: The study employed Confirmatory factor analysis (CFA) to evaluate construct validity using the four-component model established in previous research [16]. The CFA model utilized in our study encompassed the four-factor correlated model, comprising four indicators in the M&C domain, two indicators in the DB domain, three indicators in the PC domain, and two indicators in the K&U domain. Moreover, the sample size of this study was deemed sufficient to conduct a CFA analysis [63, 64].

Reliability: Data on the CAPL-2UQ was collected from a sample of 325 students to assess the test-retest reliability. Additionally, data on physical competence and daily behavior was collected from a subsample of 80 students aged 8–12. The test-retest reliability of the CAPL-2UQ was assessed at a seven-day interval after the initial administration of the test.

### Data collection procedures

This research, a segment of the broader Pakistan Initiative to Promote Physical Literacy (PAK-IPPL) study, aimed to assess physical literacy among 8-to-12-year-olds in higher secondary schools across South Punjab, Pakistan. Conducted during the 2020–2021 academic year, this study utilized the CAPL-2 under a cross-sectional framework. A randomized sampling strategy was employed to achieve a representative sample.

Ethical considerations were addressed by obtaining permission from the School education department and securing written or verbal consent from both school principals and parents of participants. The inclusion criteria for this study were as follows: (a) students without having chronic diseases and medical conditions, including cardiovascular diseases, high blood pressure, diabetes, and anemia; (b) no formal diagnosis of motor, physical, or mental disorders, which could influence study participation; and (c) students who comprehended the study requirements and exhibited a willingness to fulfill them. If participants did not meet these criteria, they were replaced with another student from the same age group and school.

A self-developed questionnaire was employed to collect data on sociodemographic and anthropometric factors, following a standardized protocol to ensure consistency and efficiency. Each school was given three days for assessment completion, with prior approval obtained from school authorities. On the first day, an introductory lecture clarified the study’s objective and guided participants in completing the questionnaire. Videos demonstrated testing procedures, and the research team addressed questions afterward. Both the self-designed and CAPL-2UQ questionnaires were administered, with support offered to participants who faced challenges in comprehension or completion. The protocol was uniformly applied across all locations to maintain consistency and accuracy.

On the second evaluation day, students completed the CAMSA and plank test under the supervision of five appraisers. Before the assessment, an assessor demonstrated and instructed the students on performing the tests. Each student was allowed one practice trial for the plank test and two for the CAMSA before commencing the three timed/scored trials. On the third day, participants underwent anthropometric measurements and the PACER test. Subsequently, students received a pedometer, instruction sheet, and daily step recording sheet, with instructions to return them after seven days. The statistical computation technique from the CAPL-2 manual was applied for missing data [60].

## Statistical analysis

In this study, IBM SPSS version 22.0 was employed for statistical analysis. Descriptive analysis calculated mean, standard deviation, and frequencies, with participants’ PL overall and domain scores determined by gender and age. Children were further categorized by PL level based on CAPL-2’s interpretive categories. Chi-squared tests, t-tests, and ANOVA determined statistical differences between variables by age and gender, while Pearson’s correlation assessed the relationship between total PL and domain. The F test and Eta-squared (η²) assessed the significance and effect size of findings, respectively, with η² values of 0.01, 0.06, and 0.14 indicating small, medium, and large effects. Statistical significance was set at *p* < 0.05. Cronbach’s alpha (α) evaluated the internal consistency reliability for each domain and subdomain construct, with values above 0.7 indicating good consistency [65]. Test-retest reliability among four domains was evaluated using Intraclass Correlation Coefficient (ICC) values and a 95% Confidence Interval [66].

Standard errors of measurement (SEM), percent error of SEM (SEM%), and minimum detectable changes (MDCs) were calculated. Confirmatory factor analysis (CFA) was performed using AmosS23 software and maximum likelihood estimation to evaluate construct validity. Fit indices, including absolute measures such as root mean square error of approximation (RMSEA), standardized root mean square residual (SRMR), and relative measures (comparative fit index [CFI] and Tucker-Lewis index [TLI]), were examined to assess model-data fit.

## Results

Table 2 displays a gender-specific anthropometric analysis for children aged 8 to 12. The average age was 10.00 years for both genders. Boys and girls exhibited similar mean heights (137.79 and 136.74, respectively), but boys had higher mean values for weight, waist circumference, and hand grip. Conversely, girls had a higher mean BMI of 16.05. The T-test revealed significant statistical differences (*P* < 0.001) among genders in waist circumference and hand grip but not in other anthropometric variables.

**Table 2:** Descriptive characteristics of the study participants

Variable	Total (*n* = 1360) Mean ± SD	Boys (*n* = 675) Mean ± SD	Girls (*n* = 685) Mean ± SD	*P*-value
Age (years)	10.00 ± 1.41	10.00 ± 1.42	10.00 ± 1.41	0.908
Height (cm)	137.26 ± 11.34	137.79 ± 11.42	136.74 ± 11.25	0.088
Weight (kg)	30.58 ± 8.62	30.82 ± 8.82	30.34 ± 8.41	0.308
BMI (kg/m2)	16.05 ± 3.16	16.04 ± 3.19	16.05 ± 3.14	0.920
WC (cm)	59.22 ± 8.74	60.35 ± 9.40	58.10 ± 7.89	< 0.001
HG	20.95 ± 7.56	21.71 ± 8.12	20.20 ± 6.89	< 0.001

*Note*: The data were presented as mean ± standard deviation. BMI: Body mass index; WC: Waist circumference; HG: Hand Grip; *P*-value < 0.05 means a significant difference

Table 3 displays the mean composite scores and reliability measures for PC, DB, K&U, and M&C domains. The retest composite scores surpassed the test composite scores overall. The PC and DB domains exhibited excellent internal consistency and reliability, with Cronbach’s alpha of 0.988 and 0.995 and ICC reliability measures of 0.979 and 0.990, respectively. Similarly, the K&U domain had a Cronbach’s alpha of 0.992 and an ICC reliability measure of 0.983, indicating excellent internal consistency and reliability. In the M&C domain, all constructs’ retest scores were higher than the test scores. Cronbach’s alpha for the M&C domain was 0.992, indicating excellent internal consistency.

**Table 3:** Test–retest reliability of the CAPL-2 in pakistani population

Variable	Test x̄ ± SD	Retest x̄ ± SD	*p*-Value^+^	Cronbach’s α	ICC (95% CI)	SEM	%SEM	MDC
PC Domain (*N* = 80)	16.42 (3.72)	16.59 (3.68)	0.668	0.988	0.979 (0.984–0.968)	0.53	3.21	1.48
DB Domain (*N* = 80)	10.56 (2.92)	10.68 (2.94)	0.007	0.995	0.990 (0.983 − 0.944)	0.29	18.85	0.81
K&U Domain (*N* = 325)	6.32 (1.94)	6.37 (1.95)	0.009	0.994	0.983 (0.978–0.986)	0.25	3.99	0.70
M&C Domain (*N* = 325)	18.56 (4.12)	18.66 (4.07)	0.031	0.990	0.980 (0.979–0.987)	0.54	2.88	1.49

*Note*: PC: Physical Competence; DB: Daily Behavior; K&U: Knowledge and Understanding; M&C: Motivation and Confidence domain; Data is presented as x̄: mean; SD: standard deviation; N/A: not applicable; Correlation: correlation is significant at the 0.01 level; Cronbach’s α: was calculated for each construct; ICC: calculated with a two way mixed model, absolute agreement and single measure; p-Value+: Paired sample t-test; ICC: < 0.02 = weak, 0.21–0.40 = fair, 0.41–0.60 = moderate, 0.61 to 0.80 = significant, and > 0.80 = excellent; SEM: standard error of measurement; %SEM: standard error of measurement as a percentage; MDC: minimum detectable change

Face and content validity: Following forward and backward translation, face and content validity were evaluated for accuracy and consistency. Experts assessed the CAPL-2UQ’s face validity in the fifth translation step, recommending modifications after pilot testing. The expert committee deemed the revised CAPL-2U questionnaire satisfactory upon incorporating these changes. To guarantee each item’s relevance, clarity, and completeness within the domains and constructs, adjustments were made. PE teachers and students rated the questionnaire’s face validity as good and satisfactory. The CAPL-2 questionnaire is provided in Appendix A. The expert committee thoroughly assessed content validity, agreeing that the final CAPL-2 Urdu translation was straightforward and comprehensible, thus ensuring its content validity.

Content validity: The Content validity ratio score of the questionnaire item is 0.89, which indicates that the questionnaire has a high level of content validity, as most of the items have been deemed to be relevant by the experts in the field. On average, 89% of the experts on the panel rated each item on the questionnaire as either “relevant” or “highly relevant” to the content area being measured.

**Fig. 3:**
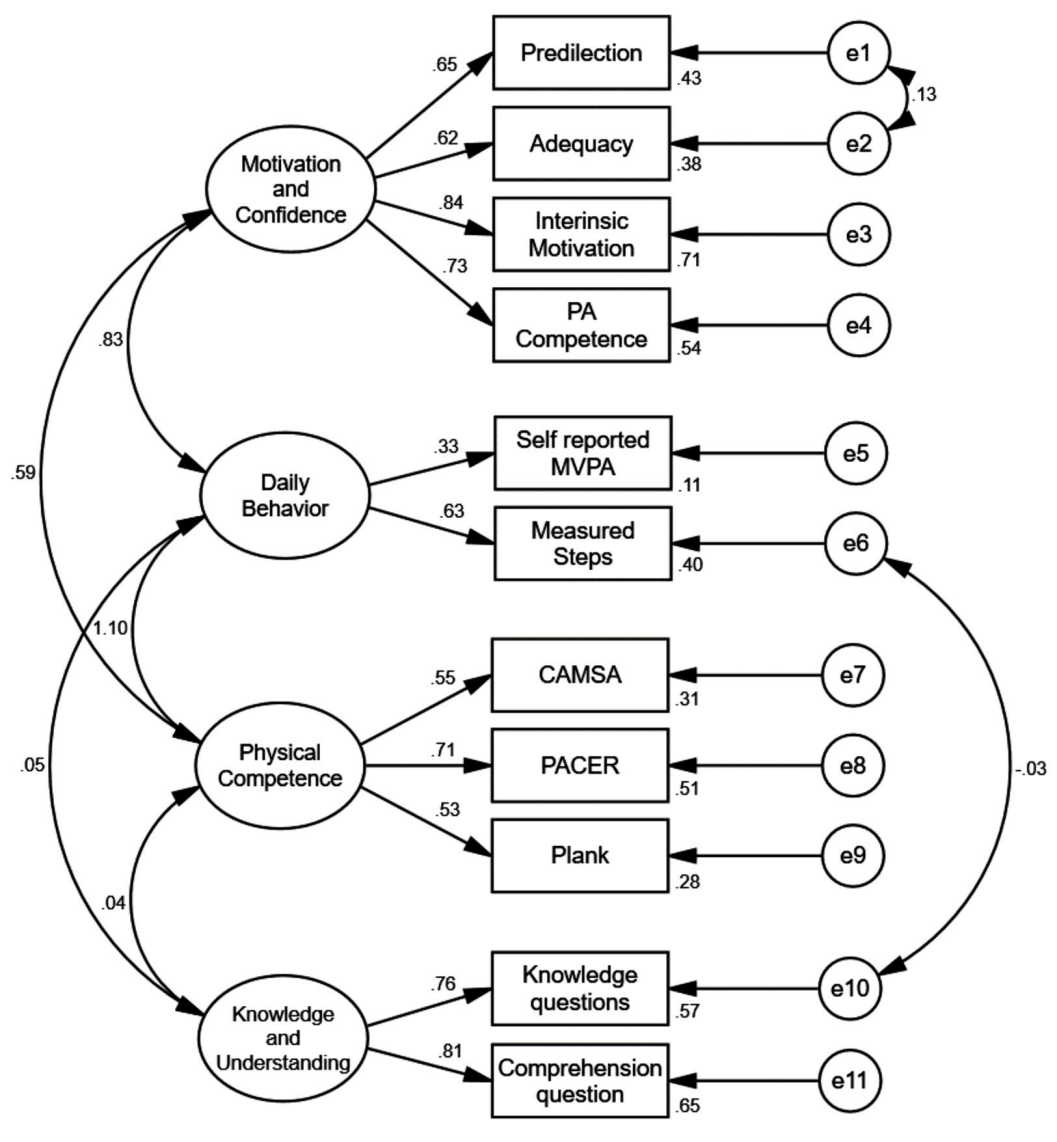
Final factor structure for Canadian assessment of physical literacy-2 with standardized factor loading

Construct validity: Fig. 3 presents the final CAPL-2 factor structure with standardized factor loadings. Although the confirmatory factor analysis yielded a chi-square statistic of 118.049 (37 degrees of freedom), suggesting poor model fit, this may be attributed to the chi-square’s sensitivity to sample size. Thus, alternative fit indices were considered. The RMSEA was a low 0.041, signifying a good fit. Similarly, the GFI and the AGFI were high at 0.984 and 0.971, respectively. The CFI, IFI, and TLI exceeded 0.97, while the NFI was slightly lower at 0.970. Collectively, these indices indicate a satisfactory model fit. Factor loadings ranged from 0.33 to 0.84, all surpassing the accepted threshold of 0.30. These loadings represent the strength of the relationship between each observed variable and the underlying factor, with higher values suggesting stronger associations.

Table 4 presents gender-specific composite scores and descriptive data for Physical Literacy and its subdomains. The average PL composite score was 51.06 out of 100. In all subdomains, boys had higher average scores than girls, except for the K&U domain, where the difference was not significant (*P* = 0.160). Notably, boys’ mean scores were significantly higher than girls in all other domains and the total score (*P* < 0.001).

**Table 4:** Gender-Specific composite scores for Physical Literacy and domains

Variable	Total (*n* = 1360) Mean ± SD	Boys (*n* = 675) Mean ± SD	Girls (*n* = 685) Mean ± SD	*P*-value	Minimum & Maximum
PL (0-100 scores)	51.06 ± 10.20	53.57 ± 9.94	48.58 ± 9.85	< 0.05	23.33–84.02
DB (0–30 scores)	12.18 ± 4.62	12.89 ± 5.22	11.47 ± 3.81	< 0.05	4.95–26.79
PC (0–30 scores)	15.95 ± 3.92	17.39 ± 3.24	14.53 ± 4.02	< 0.05	3.35–25.58
K&U (0–10 scores)	6.38 ± 1.60	6.44 ± 1.55	6.32 ± 1.63	0.160	2.97–9.90
M&C (0–30 scores)	18.02 ± 5.55	18.42 ± 5.85	17.62 ± 5.21	< 0.05	6.53–29.69

*Note*: PL: Physical Literacy; DB: Daily Behavior; PC: Physical Competence; K&U: Knowledge and Understanding; M&C: Motivation and Confidence; *P*-value < 0.05 means significant difference

Table 5 displays the CPLA-2 interpretation categories for participant distribution in physical literacy and its domains. In the composite PL scores category, 71.6% (974) of the participants were classified as “Beginning” or “Progressing,” while 28.4% (386) were classified as “Achieving” or “Excelling”. Among those in the “Beginning” and “Progressing” categories, girls accounted for 40%, whereas boys constituted 31.6%. Conversely, 18% of boys and 10.3% of girls were categorized as “Achieving” or “Excelling”. Most participants were classified as beginning and progressing in overall physical literacy and its domain scores classifications.

**Table 5:** Gender-specific summary of participants among PL and domains interpretation categories of CPLA-2

Variable	Total *N* (%)	Boys *N* (%)	Girls *N* (%)	*P*-value
PL (100 points)				
Beginning (< 41.99)	216 (15.9)	73 (5.4)	142 (10.4)	0.000
Progressing (42.00 to 54.97)	758 (55.7)	356 (26.2)	402 (29.6)	
Achieving (55.00 to 61.00)	217 (16.0)	131 (9.6)	86 (6.3)	
Excelling (> 61.01)	169 (12.4)	114 (8.4)	55 (4.0)	
DB (30 Points)				
Beginning (< 6.93)	767 (56.4)	310 (22.8)	457 (33.6)	0.000
Progressing (7.92 to 10.89)	332 (24.4)	214 (15.7)	118 (8.7)	
Achieving (10.89 to 13.86)	129 (9.5)	75 (5.5)	54 (4.0)	
Excelling (> 13.87)	131 (9.7)	75 (5.6)	56 (4.1)	
PC (30 points)				
Beginning (< 12.09)	251 (18.5)	63 (4.6)	188 (13.8)	0.000
Progressing (12.10 to 17.80)	680 (50.0)	322 (23.7)	358 (26.3)	
Achieving (17.81 to 20.04)	246 (18.1)	154 (11.3)	92 (6.8)	
Excelling (> 20.05)	183 (13.5)	136 (10.0)	47 (3.5)	
K&U (10 scores)				
Beginning (< 4.95)	216 (15.9)	93 (6.8)	123 (9.0)	< 0.05
Progressing (4.95 to 6.93)	763 (56.1)	387 (28.5)	376 (27.6)	
Achieving (6.94 to 7.92)	264 (19.4)	145 (10.7)	119 (8.8)	
Excelling (> 7.92)	117 (8.6)	50 (3.7)	67 (4.9)	
M&C (30 scores)				
Beginning (< 12.77)	226 (16.6)	124 (9.1)	102 (7.5)	0.000
Progressing (12.77 to 20.68)	658 (44.8)	305 (22.4)	353 (26.0)	
Achieving (20.69 to 24.64)	312 (22.9)	143 (10.5)	169 (12.4)	
Excelling (> 24.64)	164 (12.1)	103 (7.6)	61 (4.5)	

*Note*: PL: Physical Literacy; DB: Daily Behavior; PC: Physical Competence; K&U: Knowledge and Understanding; M&C: Motivation and Confidence; *P*-value < 0.05 means significant difference

Table 6 presents the correlations between Physical Literacy and its domain composite scores using Pearson correlation analysis. The strongest relationships with Physical Literacy were observed among the DB, PC, and M&C domains, with correlation coefficients of 0.766, 0.785, and 0.844, respectively. Moderate correlations were noted between DB and PC (0.581), M&C and PC (0.513), and PC and M&C (0.450). Conversely, the weakest correlations involve K&U with composite PL and the other three domains, with coefficients of 0.174, 0.021, 0.032, and 0.002, respectively.

**Table 6:** Correlation among physical literacy and four subdomains according to CAPL-2

	PL	DB	PC	K&U
PL	1			
DB	0.766**	1		
PC	0.785**	0.581**	1	
K&U	0.174**	0.021	0.032	1
M&C	0.844**	0.513**	0.450**	0.002

*Note*: Physical Literacy; DB: Daily Behavior; PC: Physical Competence; K&U: Knowledge and Understanding; M&C: Motivation and Confidence; ** Correlation is significant at the 0.01 level (2-tailed)

Table 7 presents age and gender-specific analyses of PL and its domains. ANOVA tests evaluated age and gender effects on total PL scores and the four domains (DB, PC, K&U, and M&C). Results demonstrated significant age (F (4,1360) = 6.929, *p* < 0.01, η2 = 0.021, small effect size) and gender (F (1,1360) = 87.761, *p* < 0.01, η2 = 0.062, medium effect size) impacts on PL scores, alongside an interaction effect. Boys scored higher than girls, with a significant statistical difference (*p* < 0.001). Total PL scores increased with age for both genders. For the DB domain, gender significantly affected PL scores (F (1,1350) = 69.140, *p* < 0.01, η2 = 0.049, small effect size), while age did not (F (4,1350) = 1.264, *p* = 0.282, η2 = 0.004, no effect size), an interaction effect was observed. Boys outperformed girls at all ages, with a significant difference (*p* < 0.001). Scores increased with age for both genders, except for 11-year-old boys and 12-year-old girls, where they declined. In the PC domain, age (F (4,1350) = 12.199, *p* < 0.01, η2 = 0.035, small effect) and gender (F (1,1350) = 216.216, *p* < 0.01, η2 = 0.138, large effect) significantly influenced PL scores, with an interaction effect. Boys had higher scores than girls across all ages, with a significant difference (*p* < 0.001). Scores increased with age for both genders.

The K&U domain results demonstrated a significant age effect on total PL scores (F (4, 1350) = 4.035, *p* < 0.05, η2 = 0.012, small effect size) but no significant gender effect (F (1, 1350) = 1.996, *p* = 0.158, η2 = 0.001, small effect size), with an interaction effect observed. Boys surpassed girls in all age groups, except among 12-year-olds, where girls scored higher; however, only the 12-year-old group showed a significant gender difference (*p* > 0.05). In the M&C domain, age and gender significantly impacted total PL scores (F (4,1350) = 2.724, *p* < 0.05, η2 = 0.008, small effect size; F (1,1350) = 7.136, *p* < 0.05, η2 = 0.005, small effect size, respectively). Boys (18.43) scored higher than girls (17.63), with the gender difference being significant (*p* < 0.05), and scores increased with age for both genders.

**Table 7:** Age and gender-specific scores for physical literacy and domains

Variable		8 years		9 years		10 years		11 years		12 years	
		Boys	Girls	Boys	Girls	Boys	Girls	Boys	Girls	Boys	Girls
		Mean ± SD	Mean ± SD	Mean ± SD	Mean ± SD	Mean ± SD	Mean ± SD	Mean ± SD	Mean ± SD	Mean ± SD	Mean ± SD
Physical Literacy		52.13 ± 7.96	47.70 ± 7.62	54.24 ± 9.80	48.93 ± 8.75	55.51 ± 13.61	49.23 ± 10.61	56.04 ± 14.28	51.39 ± 12.51	58.24 ± 13.43	51.91 ± 12.57
	**Total DB (30 points)**	11.67 ± 2.66	10.71 ± 1.20	12.41 ± 4.23	11.17 ± 2.84	13.31 ± 5.72	11.50 ± 3.90	13.59 ± 5.94	12.12 ± 4.77	13.50 ± 6.42	11.84 ± 4.96
Daily Behavior	Weekly average steps scores	7.57 ± 2.48	7.04 ± 0.67	8.24 ± 3.98	7.39 ± 2.50	9.35 ± 5.28	7.64 ± 3.66	9.41 ± 5.58	8.51 ± 4.38	9.55 ± 6.02	8.18 ± 4.61
	Self-Reported MVPA days	5.23 ± 1.11	4.77 ± 1.05	5.33 ± 1.13	4.85 ± 1.21	5.09 ± 1.15	4.96 ± 1.12	5.22 ± 0.91	4.64 ± 1.02	4.99 ± 1.00	4.75 ± 1.04
	**Total PC (30 points)**	16.29 ± 3.21	13.62 ± 4.34	17.13 ± 3.57	13.99 ± 4.91	17.33 ± 2.82	14.80 ± 3.20	17.47 ± 3.07	14.96 ± 3.72	18.73 ± 3.01	15.25 ± 3.51
Physical Competence	CAMSA Scores	12.67 ± 2.71	10.47 ± 2.74	12.62 ± 2.71	10.87 ± 3.40	13.11 ± 2.53	10.85 ± 2.16	13.17 ± 2.89	11.39 ± 2.63	13.88 ± 3.09	11.42 ± 2.80
	PACER (20 m) scores	3.67 ± 1.37	2.82 ± 1.59	4.12 ± 1.64	2.78 ± 1.73	4.04 ± 1.44	3.20 ± 1.36	4.24 ± 1.60	3.18 ± 1.44	4.62 ± 1.37	3.20 ± 1.56
	Plank (sec) scores	8.10 ± 2.48	7.05 ± 3.00	8.50 ± 2.00	7.33 ± 3.16	8.61 ± 1.86	7.72 ± 2.38	8.53 ± 1.93	7.72 ± 2.51	9.15 ± 1.49	7.98 ± 2.41
	**Total K&U (10 points)**	6.13 ± 1.49	6.07 ± 1.75	6.34 ± 1.56	6.28 ± 1.59	6.59 ± 1.44	6.19 ± 1.60	6.54 ± 1.66	6.36 ± 1.81	6.58 ± 1.60	6.68 ± 1.35
Knowledge and Understanding	Knowledge Quiz Items	2.65 ± 0.56	2.60 ± 0.60	2.73 ± 0.55	2.69 ± 0.59	2.76 ± 0.52	2.67 ± 0.62	2.76 ± 0.58	2.74 ± 0.72	2.79 ± 0.59	2.79 ± 0.60
	PA comprehension	3.48 ± 1.11	3.47 ± 1.28	3.61 ± 1.16	3.60 ± 1.15	3.83 ± 1.06	3.52 ± 1.18	3.78 ± 1.23	3.62 ± 1.21	3.79 ± 1.15	3.89 ± 0.99
	**Total M&C (30 points)**	17.96 ± 5.88	17.32 ± 5.40	18.19 ± 5.86	17.22 ± 5.49	18.21 ± 5.80	17.03 ± 4.91	18.44 ± 6.24	18.07 ± 4.94	19.31 ± 5.44	18.48 ± 5.21
Motivation and Confidence	Predilection Score	4.55 ± 1.95	4.23 ± 1.88	4.57 ± 1.87	4.34 ± 1.86	4.66 ± 1.96	4.17 ± 1.87	4.73 ± 1.94	4.82 ± 1.80	4.81 ± 1.90	4.57 ± 1.91
	Adequacy Score	4.63 ± 1.93	4.59 ± 1.86	4.68 ± 1.83	4.57 ± 1.77	4.82 ± 1.81	4.45 ± 1.70	4.74 ± 1.92	4.66 ± 1.79	5.30 ± 1.65	4.77 ± 1.75
	Intrinsic Motivation	4.38 ± 1.72	4.22 ± 1.54	4.47 ± 1.81	4.09 ± 1.63	4.40 ± 1.80	4.12 ± 1.58	4.53 ± 1.99	4.20 ± 1.51	4.64 ± 1.58	4.61 ± 1.56
	Physical Competence	4.40 ± 1.69	4.28 ± 1.62	4.47 ± 1.70	4.23 ± 1.64	4.33 ± 1.51	4.28 ± 1.37	4.43 ± 1.72	4.38 ± 1.51	4.57 ± 1.45	4.52 ± 1.58

## Discussion

The current study is the first study that aimed to translate the CAPL-2 into Urdu language and cross-culturally adapt to the Pakistani context, ensuring its reliability and validity in measuring PL in 8–12-year-old Pakistani children. Additionally, the study aimed to provide the PL status of school children from South Punjab, Pakistan, stratified by age, gender and CAPL-2 interpretative categories. The study assessed the test-retest reliability of CAPL-2U over a one-week interval, identifying excellent internal consistency across all four domains. This aligns with findings from a prior Spanish study [50], highlighting CAPL-2U’s reliability for assessing Pakistani children’s PL level. Other studies in Canada [67], Denmark [16], and China [49] reported good internal consistency for the M&C domain. The Chinese study specifically reported an internal consistency reliability of 0.82, indicating good reliability for the M&C domain. However, for the K&U domains, Cronbach’s alpha was 0.52, indicating poor reliability. In contrast, our study demonstrated better results, with Cronbach’s alpha ranging from 0.994 to 0.990 for both domains.

Regarding construct validity, the CFA analysis based on a sample of 1360 Pakistani students suggests that the CAPL-2U four-factor model used in the analysis fits the data well. The four-factor was based on the original CAPL-2 model [48]. The CFA model fit indices suggest that the CAPL-2U is a valid tool for evaluating PL in Pakistani school children aged 8–12. The CAPL-2U maintains the original CAPL-2’s four-factor model in line with the PL theoretical framework. Unlike the Chinese validation studies that eliminated some indicators, the CAPL-2U did not drop any indicators. The Chinese study dropped two indicators from the K&U and predilection scale to adjust the model and to achieve model fit [49]. The current study’s four-factor model was similar to the Danish study [16], which retained all constructs and reported a good model fit. These results indicate that the CAPL-2U is a reliable and valid tool for measuring PL in the target population. The success of the validation process may be attributed to maintaining consistency with the original model and avoiding unnecessary modifications.

Although CAPL-2 model fits may not significantly differ across various translation studies, cultural and linguistic differences among populations can result in variations that should be acknowledged. The current study achieved a better model fit by including all constructs and items and having a larger sample size than the Chinese and Greek studies [17, 49]. Quality data was collected due to students’ interest and willingness to participate and complete tests. Therefore, adaptations to the CAPL-2 tool must account for these variations when evaluating its effectiveness in diverse environments. Acknowledging these factors can help researchers ensure a coherent and comprehensive approach to their research, enhancing its academic credibility and rationale.

The correlation results indicate that DB, PC, and M&C domains strongly correlate with total PL. Most domains show a significant and positive correlation, except the K&U domain, which has a weak, nonsignificant correlation coefficient with total PL and the other three domains. These correlation results align with previous validation studies showing a significant and positive correlation among most domains [16, 49]. However, the K&U domain has a weak correlation with total PL, similar to a Chinese study that also reported a nonsignificant and weak correlation [49]. In contrast, a Danish study reported no correlation between K&U and M&C domains [16]. The current study sample size is larger than the previous validation studies; for this reason, the result of the present study correlation is more significant than previous studies [16, 49].

Regarding the status of PL and domain scores, a significant gender difference is observed in the current study, with boys scoring higher than girls in total PL and the other four subdomains with significant differences, except for the K&U, where the difference was nonsignificant. A Chinese study found that boys outperformed girls in all areas except K&U, but this cannot be applied to the Pakistani population [49]. The difference in results may be due to cultural and societal factors favoring boys, such as better exposure to sports and related knowledge [68]. Conversely, girls in Pakistan have limited sports participation [69], leading to a lack of knowledge regarding cardiovascular health, recommended physical activity time, muscular endurance, and sports performance improvement, as they solely concentrate on academics. It is also crucial to consider the sampled population since the CAPL-2 scoring is based on Canadian normative data [49]. The current study’s sample size was larger than other validation studies [16, 17, 49], and Pakistani boys and girls differed from those in other population studies, resulting in score differences. Future studies should examine the impact of sample size and participant demographics on assessment scores and strive to make the sample comparable to the Canadian study. In Pakistani children, the overall PL increases significantly (*P* = 0.05) with age, and this trend is consistent across genders. Specifically, boys tend to score higher than girls. These findings align with previous studies [17, 42, 49].

Further, the analysis regarding the status of DB indicates a trend in physical activity among boys and girls within a particular age range. Boys exhibit an increase in PA from ages 8 to 10, followed by a decrease at age 11, and then an increase again. Conversely, girls exhibit increased PA as they age, but a decline is observed at age 12. Overall, boys achieved higher scores than girls, and the results are similar to previous studies [16, 17]. This trend in PA could have multiple interpretations. Boys’ increased PA at ages 8–10 could be attributed to their interest in physical play and sports. The subsequent decline at age 11 may be due to academic or social interests or limited opportunities for PA. With the limited literature on female PA in Pakistan, we concluded that girls’ PA increased during this period due to their interest and permission to play, as cultural and religious norms in Pakistan allow girls to participate in sports at this age [68]. However, the decrease in PA at age 12 is likely to be linked to puberty-related changes [70]. Cultural and religious norms and restrictions may limit girls’ sports and physical activities after puberty in Pakistan [71].

Further, the PC scores increased as age advanced with significant (*P* = 0.05) gender and age difference, and this trend is consistent across genders [17]. Based on the results, M&C scores were higher in boys than girls, with a significant difference (*P* = 0.05). Additionally, there was a noticeable trend in M&U scores concerning age. M&U scores in boys increased as they grew older, while girls’ M&U scores followed a different pattern. Girls’ M&U scores increased from ages 8 to 9, decreased at age 10, and then increased again from ages 11 to 12. The observed trend in boys’ M&U scores could be due to their participation in sports and physical activity.

On the other hand, our study suggests that cultural and religious norms lead to reduced physical activity and sports involvement among girls as they approach puberty [68, 72]. As a result, girls’ M&C scores decrease as they grow older, a similar trend was observed in an Iranian study [51]. The data also indicates that girls are more motivated to participate in sports during their early years. The K&U scores showed a significant (*P* = 0.05) increase with age, consistent with previous research [17, 49]. Boys generally scored higher than girls, except for 12-year-olds, which contradicts the findings of a Greek study where only 8-year-old girls scored higher [17]. This difference warrants further investigation.

The PL interpretative categories results indicate that 71.6% of children in South Punjab are in the beginning or progressing stage, while only 28.4% are achieving or excelling. These results are superior to those of a Chinese study in which 98.8% of children were in the beginning or progressing stage [49]. The disparity may be due to the use of an accelerometer rather than the suggested pedometer in the Chinese study [49]. Additionally, an Iranian study found that only 11.4% of 8 to 12-year-old children were achieving or excelling, which is lower than the present study’s findings [51]. The reliability and construct validity analysis results demonstrate that the CAPL-2U’s translation and cross-cultural adaptation were successfully established, affirming its validity for assessing PL in the Pakistani population. Based on this evidence, we recommend that researchers formulate intervention guidelines to integrate PL into curriculum and practice, thereby promoting children’s health and well-being.

### Strength and novelty:

This study presents several strengths and novel contributions that enhance its validity and generalizability. Notably, it introduces the first translation and cross-cultural adaptation of CAPL-2 into Urdu, specifically tailored for the Pakistani context. This advancement addresses a significant gap in the literature by providing a reliable PL assessment tool for use in Pakistan and potentially across South Asia. A notable feature of this research is the employment of a larger sample size than those used in previous validations, thereby increasing the study’s statistical power. The precision in data collection was ensured by a trained team, which minimized biases and enhanced the accuracy of the findings.

Furthermore, the use of a pedometer to estimate physical activity represents a more objective measure than self-reported data, lending additional reliability to our results and facilitating comparisons with international studies. The adoption of stratified random sampling improved the study’s representativeness and generalizability, while a high response rate further reduced potential biases. To address missing data, replacements were drawn from same schools and age groups, and statistical data imputation techniques were utilized to mitigate any impact on the study’s outcomes. Collectively, these methodological strengths underscore the robustness and importance of our findings.

### Applicability:

The applicability of this study’s findings significantly advances the promotion of Physical Literacy in Pakistan. By successfully translating and culturally adapting the CAPL-2 into Urdu, we have introduced a pivotal tool for PL assessment in the region. This adaptation not only offers a valid instrument for gauging PL but also extends its utility to Urdu-speaking populations in South Asia, particularly in Pakistan and India. The Urdu version of CAPL-2 is designed to be both concise and user-friendly, facilitating its application in diverse environments such as schools, community centers, and clinics. Its versatility ensures inclusivity for children from various socioeconomic, ethnic, and religious backgrounds.

Moreover, including age and gender differences in the study results offers more profound insights into PL variations. Addressing these disparities enables equitable opportunities, shaping tailored PL programs through informed policymaking and education strategies. Prospective longitudinal studies will amplify this tool’s utility, paving the way for healthier futures among Pakistani children.

### Limitations and future directions:

Although the study provides valuable insights, some limitations must be acknowledged. Specifically, the study did not evaluate PL’s convergent validity with other tools like PLAY and Canadian Sports for Life, limiting the generalizability of the findings. To address this limitation, future researchers should consider using a larger sample size across Pakistan to assess other types of reliability and validity. While our findings rely on a cross-sectional design, subsequent studies, especially longitudinal ones, are essential to explore deeper implications for promoting PL in Pakistan. As we lay a foundational platform for studying PL in Pakistan, future research should emphasize further validation of Urdu CAPL-2, devise strategies to enhance PL and investigate its lasting impact on health. This continuous exploration promises to equip children with the vital PL skills for healthier, more active lives.

## Conclusion

This study is first study to adapt the CAPL-2 for use in Pakistan, demonstrating its reliability and validity for assessing physical literacy among Pakistani children aged 8–12. The successful translation into Urdu and adaptation to Pakistani context are supported by confirmatory factor analysis, which confirms the model’s fit, thus establishing the instrument’s validity. The CAPL-2 Pakistani version exhibits high internal consistency and test-retest reliability across all domains, underlining its robustness as a tool for comprehensive PL assessment.

Findings from South Punjab highlight a critical need for targeted PL interventions, given the region’s current PL levels ranging from Beginning to Progressing stages. The validated CAPL-2 Pakistani version is instrumental in facilitating detailed assessments and shaping interventions aimed at fostering active and healthy lifestyle choices from a young age. Future research should focus on developing and implementing longitudinal, PL-centered interventions to improve the PL and overall health of Pakistani children.

### Electronic supplementary material

Below is the link to the electronic supplementary material.

Supplementary Material 1

## Data Availability

Data can be requested by contacting the corresponding author on a reasonable request.
