# Supplementary material for: Assessment of physical literacy in 8- to 12-year-old Pakistani school children: reliability and cross-validation of the Canadian assessment of physical literacy-2 (CAPL-2) in South Punjab, Pakistan
Source: BMC Public Health. 2024 Jun 28;24:1726. doi: 10.1186/s12889-024-19185-3 (PMC11212239; doi:10.1186/s12889-024-19185-3)
Supplement: Supplementary file 1 — Supplementary Material 1 [file 12889_2024_19185_MOESM1_ESM.pdf]

CAPL–2 Questionnaire

What Do You Think About Physical Activity?

When we ask you about physical activity, we mean when you are moving around, playing, or exercising. *Physical activity* is any activity that makes your heart beat faster or makes you get out of breath some of the time.

Why are we asking you these questions?

We want to know what kids, like you, think about physical activity, sports, and exercise.

Please Remember:

- There are no right or wrong answers! We only want to know what you think.
- If you do not know an answer, please write your best guess.
- There is no time limit, so please take all of the time you need.

What's Most Like Me?

For each question, you have to read two sentences and then circle the sentence you think is **MORE LIKE YOU.**

Try the following **SAMPLE QUESTION:**

**Some kids have one nose on their face BUT Other kids have three noses on their face**

That shouldn't be too hard for you to decide!

Once you have circled the sentence that is <u>more like</u> you, then you have to decide if it is **REALLY TRUE** for you or **SORT OF TRUE** for you.

Here is another sample question for you to try. Remember, to answer the question you need to do two things:

1) **<u>First, circle the sentence that is more like you.</u>**

2) **<u>Then, put a check in the correct box if it is REALLY TRUE or SORT OF TRUE for you.</u>**

THERE ARE NO RIGHT OR WRONG ANSWERS, JUST TELL US WHAT YOU THINK IS **MOST LIKE YOU!**

Sample Question #2

**Some kids like to play with computers**	**BUT**	**Other kids don't like playing with computers**	
**□ REALLY TRUE for me**	**□ SORT OF TRUE for me**	**□ REALLY TRUE for me**	**□ SORT OF TRUE for me**

Now you are ready to start filling in this form. **Remember, in each box you need to <u>circle</u> what is most like you and then check a box for "really" or "sort of" true.** Take your time and do the whole form carefully. If you have questions, just ask! If you think you are ready you can start now.

BE SURE TO FILL IN EACH PAGE!

What's Most Like Me?

**Some kids don't like playing active games**	**BUT**	**Other kids really like playing active games**
**□ REALLY TRUE for me** / **□ SORT OF TRUE for me**		**□ REALLY TRUE for me** / **□ SORT OF TRUE for me**

**Some kids are good at active games**	**BUT**	**Other kids find active games hard to play**
**□ REALLY TRUE for me** / **□ SORT OF TRUE for me**		**□ REALLY TRUE for me** / **□ SORT OF TRUE for me**

**Some kids don't have much fun playing sports**	**BUT**	**Other kids have a good time playing sports**
**□ REALLY TRUE for me** / **□ SORT OF TRUE for me**		**□ REALLY TRUE for me** / **□ SORT OF TRUE for me**

**Some kids do well in most sports**	**BUT**	**Other kids feel they aren't good at sports**
**□ REALLY TRUE for me** / **□ SORT OF TRUE for me**		**□ REALLY TRUE for me** / **□ SORT OF TRUE for me**

**Some kids don't like playing sports**	**BUT**	**Other kids really enjoy playing sports**
**□ REALLY TRUE for me** / **□ SORT OF TRUE for me**		**□ REALLY TRUE for me** / **□ SORT OF TRUE for me**

**Some kids learn to play active games easily**	**BUT**	**Other kids find it hard learning to play active games**
**□ REALLY TRUE for me** / **□ SORT OF TRUE for me**		**□ REALLY TRUE for me** / **□ SORT OF TRUE for me**

Thank you for telling us which kids are most like you!

We just have a few more questions. Please turn to the next page.

Why are you active?

Boys and girls can be **active** by doing all sorts of things:

- Exercise (walking, keeping fit, or gym class)
- Playing outside or doing active things (like playing in the park)
- Sports (like soccer, tennis, hockey, dance or swimming)

Below are some reasons why you might be active.

Please read each sentence and tell us <u>how true it is</u> for you.

**I am active because...**					
	Not true for me	Not really true for me	Sometimes true for me	Often true for me	Very true for me
being active is fun	☐	☐	☐	☐	☐
I enjoy being active	☐	☐	☐	☐	☐
I like being active	☐	☐	☐	☐	☐

How do you feel about being active?

The next section has some sentences describing how girls and boys feel about BEING ACTIVE and DOING ACTIVE THINGS (like active games, playing outside and doing sports).

Please read each sentence and tell us <u>how much each sentence is like you.</u>

	Not like me at all	Not really like me	Sometimes like me	Quite a lot like me	Really like me
When it comes to playing active games, I think I am pretty good.	☐	☐	☐	☐	☐
I think I do well at activities compared to other children	☐	☐	☐	☐	☐
When it comes to being active, I have good skills.	☐	☐	☐	☐	☐

What do you know about physical activity?

<u>Please circle only one answer for each question</u>

1. How many minutes each day should you and other children do physical activities that make your heart beat faster and make you breathe faster, like walking fast or running? Count the time you should be active at school and also when you are at home or in your neighbourhood.

a) 20 minutes

b) 30 minutes

c) 60 minutes or 1 hour

d) 120 minutes or 2 hours

2. There are many different kinds of fitness. One type is called endurance fitness, or aerobic fitness, or cardiorespiratory fitness. Cardiorespiratory fitness means:

a) How well the muscles can push, pull, or stretch
b) How well the heart can pump blood and the lungs can provide oxygen
c) Having a healthy weight for our height
d) Our ability to do sports that we like

3. Muscular strength or muscular endurance means:

a) How well the muscles can push, pull, or stretch
b) How well the heart can pump blood and the lungs can provide oxygen
c) Having a healthy weight for our height
d) Our ability to do sports that we like

4. If you wanted to GET BETTER AT A SPORT SKILL (like kicking and catching a ball), what would be the best thing to do?

a) Read a book about kicking and catching a ball

b) Wait until you get older

c) Try exercising or being more active

d) Watch a video, take a lesson, or have a coach teach you how to kick and catch

5. This story about Sally is missing some words. Choose from the words in the box to fill in the missing words in the story. Each word can only be used to fill one blank space in the story. There are more words than blank spaces, so not all words will be used.

Fun	Stretches	Endurance	
Pulse	Breathing	Flexibility	Good
Strength	Bad	Sport	

Sally tries to be active every day. Running every day is good for her heart and her lungs. Sally thinks that physical activity is ____________and is also______________for her. At her sport team's practice she does more running to improve her_______________. The team also does exercises like push-ups and sit-ups that increase her____________. When cooling down, she______________ to improve her flexibility and slow her heart rate. After exercising, she checks her heart rate which is also called a___________.

6. During the past week (7 days), on how many days were you physically active for a total of at least 60 minutes per day? Count all of the time you spent doing activities that increase your heart rate or made you breathe hard.

I was active for 0 1 2 3 4 5 6 7 days

Tell us about yourself!

Please circle one number, or word, or choice for each question

What school grade are you in?

If you are not in school today, please circle the grade you will be in on the next day that you will go to school.

0 1 2 3 4 5 6

Are you a:

Boy Girl

What month is your birthday in?

Jan Feb Mar Apr May Jun Jul Aug Sep Oct Nov Dec

How old are you?

5 6 7 8 9 10 11 12 13 14 15

Thank you for answering our questions!

☺
